# The efficacy of fractional CO_2_ laser with or without triamcinolone acetonide or 5-fluorouracil in the treatment of early postburn hypertrophic scars

**DOI:** 10.1007/s10103-024-04256-z

**Published:** 2025-01-23

**Authors:** Bassel Younes, Elsayed Mandour, Mohammed Soliman Hashish, Tarek Gamal Shoukr

**Affiliations:** 1 Plastic Surgery Department, Faculty of Medicine, Tanta University, Tanta, Egypt; 2 General Surgery Department, Faculty of Medicine, Tanta University, Tanta, Egypt

**Keywords:** Fractional CO_2_ laser, Triamcinolone acetonide, 5-Fluorouracil, Postburn, Hypertrophic scars

## Abstract

Hypertrophic scars (HTSs) are the result of an abnormal healing process resulting from burns and other severe traumas. The symptoms of that condition include skin irritation, discomfort, and itching. This study aimed to assess the efficacy of fractional carbon dioxide (CO_2_) laser therapy alone or with triamcinolone or 5-fluorouracil (FU) in the treatment of early post-burn hypertrophic scars (HTSs) that develop during the first 6 months after the injury. A prospective, randomized, single-blind comparative study was conducted on 30 patients aged 16–65 with hypertrophic scars (HTS) resulting from burns. Patients had no prior treatment for their scars. We randomly assigned participants to one of three groups: Group A received fractional CO_2_ laser therapy alone, Group B received fractional CO_2_ laser therapy with topical 5-fluorouracil, and Group C received fractional CO_2_ laser therapy with topical triamcinolone acetonide. All treatment groups showed significant improvements (*p* < 0.05) in overall scar severity and height. Patients in Group C (fractional CO_2_ laser + triamcinolone) demonstrated significant improvements in scar pliability, height, and pigmentation (*p* < 0.05). In contrast, patients in Group B (fractional CO_2_ laser + 5-FU) showed significant reductions in scar vascularity, pliability, and height following treatment (*p* < 0.05). While all groups reported minor changes in pain and itching, there were no significant differences in these symptoms between Group B and Group C. HTSs of this trial revealed reductions in overall scar surface area and thickness and improvement of pliability and pigmentation; however, there was not statistically significant difference between the effect of 5-fluorouracil and triamcinolone acetonide (TAC), suggesting that neither drug offers better efficacy over the other. Level I, singleblinded randomized control study.

## Introduction

Hypertrophic scars (HTSs) refer to an abnormal healing process of burns and traumatic injuries, resulting in symptoms such as itching, pain, and skin irritation. Despite the progress made in burn therapy, care, and rehabilitation, the presence of scars that cause dysfunction and deformity continues to be a significant challenge in clinical practice. However, these disorders themselves do not pose a direct threat to one’s health [1].

Various treatment options are currently available for visible hypertrophic burn scars. These options include applying silicone sheets, using pressure dressings, administering radiation, cryotherapy, performing surgical procedures, and using local or systemic pharmacotherapy agents such as 5-fluorouracil (FU), bleomycin, analgesics, corticosteroids, and antihistamines [2].

The application of ablative 10,600-nm fractional carbon dioxide (CO_2_) laser treatment, which operates on the principle of fractional photo thermolysis, has been employed to enhance the aesthetic and functional aspects of scars, yielding varying levels of achievement [3].

Ablative fractional lasers create microthermal treatment zones that penetrate deeply and stimulate collagen remodeling, leading to subsequent dermal regeneration. Moreover, the tissue around each treatment zone undergoes accelerated re-epithelization, leading to enhanced wound healing compared to conventional CO_2_ ablation [4].

Medical professionals have highlighted the significance of promptly and actively treating hypertrophic scars to prevent their development. Safra et al. demonstrated that applying CO_2_ fractional laser treatment shortly after surgery for scars was both safe and efficacious [5].

5-FU is a pyrimidine analog that is classified as an anti-cancer medication. It works by inhibiting the action of thymidylate synthetase, which reduces the amount of DNA and RNA produced typically. To be more specific, it specifically targets cells that are highly proliferative and metabolically active. For example, it targets fibroblasts in skin wounds that are responsible for excessive collagen formation. Furthermore, it suppresses the transforming growth factor (TGF)-b1 signaling pathway, essential in producing collagen I. This pathway is implicated in many processes. Each one of these effects is accomplished without producing necrosis of the tissue [6].

Intralesional steroid injections have been given the green light by the International Advisory Panel on Scar Management as a therapy option for HTSs [7].

Studies have shown that corticosteroids can lead to the regression of keloids through various mechanisms. Due to the administration of triamcinolone acetonide (TAC), the quantities of alpha-1-antitrypsin and alpha-2-macroglobulin present within the body are significantly decreased. Typically, these levels are present in higher concentrations in keloidal tissue and act as natural inhibitors of collagenase in the human skin. Keloidal tissue is a type of tissue that is seen in humans. This is number 24. Corticosteroids impact fibroblasts’ growth and production abilities, leading to their deterioration. In addition, scar tissues that were subjected to methylprednisolone treatment demonstrated decreased levels of TGF-β, insulin-like growth factor-1 (IGF-1), and hydroxyproline [8, 9]. TAC is the most frequently utilized corticosteroid for treating keloids [10].

This research’s purpose was to investigate the effects of combining fractional CO_2_ laser treatment with either triamcinolone acetonide or 5-fluorouracil on scar remodelling and clinical outcomes in individuals diagnosed with early postburn HTSs. This was achieved through a randomized control study.

## Methods

Thirty patients aged 16–65 with previously untreated hypertrophic scars (HTS) resulting from burn injuries participated in this study, which was conducted between January 2023 and January 2024. Exclusion criteria included pregnancy, lactation, active infections, connective tissue disorders, lidocaine allergy, systemic diseases affecting the treatment area, oral retinoid use within the past six months, large-area scars, severe contractures, suspected malignancy, and unrealistic expectations.

Patients were randomly assigned to one of three groups utilizing a lottery system, which included labeling the groups as Group A, Group B, and Group C and creating identical slips of paper, one for each participant. On each slip, we wrote one of the group labels (e.g., “A,” “B,” or “C”). The total number of slips corresponds to the total number of participants and maintains equal representation (30 participants = 10 slips for each group). We folded all the slips to make them appear identical, placed them into a bag, and mixed them thoroughly to ensure randomness. Each participant was asked to draw one slip from the bag, and their ID was recorded for their assigned group to ensure proper tracking.

Group A received fractional CO_2_ laser treatment alone; Groups B and C had the same laser treatment paired with topical 5-fluorouracil and triamcinolone acetonide, respectively. Every participant got a full baseline assessment, which included digital scar photography, physical examination, and medical history. Scar characteristics and patient satisfaction at every visit were assessed using the Vancouver Scar Scale (VSS) and Patient Scar Assessment Scale. Patients completed their six therapy sessions at one-month intervals till January 2024. Clinical information collected covered demographics, burn injury features, VSS scores, scar appearance, and laser treatment parameters.

All participants received a topical anesthetic cream containing lidocaine and prilocaine applied one hour before laser treatment. The entire scar was treated with a fractional ablative CO_2_ laser (SmartXide DOT, DEKA, Italy). Laser parameters included a power range of 15–20 W (Stack 3 or 4), a dwell time of 700–1000 microseconds, and a microchannel spacing of 500–700 micrometers. The parameters difference was divided into two categories according to scar height as scar height < 5 mm received the low parameters as a power of 15 (Stack 3), a dwell time of 700 microseconds, a microchannel spacing of 500 micrometers, a density of 13.56% and the depth of the ablated channels is estimated to be between 200 micrometers to 400 micrometers or > 5 mm received the high parameters as a power of 20 W (Stack 4), a dwell time of 1000 microseconds, a microchannel spacing of 700 micrometers, a density of 9.24% and the depth of the ablated channels is estimated to be between 400 micrometers to 600 micrometers.

Such depths are effective for scar treatment and drug delivery into the dermis and deeper layers.

Immediately following laser treatment, participants received either distilled water (Group A), 50 mg/mL 5-fluorouracil solution at a dose of 5 mg/cm^2^ with a volume ranging from 1 to 2 ml based on the surface area in group B, or 40 mg/mL triamcinolone acetonide solution at a dose of 4 mg/cm^2^ with a volume ranging from 0.5 to 2 ml based on the surface area in group C applied topically. It was rubbed until it was equally distributed, usually about a minute. The surface area of the scars was within 20 cm^2^ to be similarly distributed. Treatment allocation was blinded. Patients with two scars received different treatments for each scar, as was on one patient, and the lottery slip of the first scar was group A, and the second was group B. Six treatment sessions were administered at one-month intervals.

**Fig. 1:**
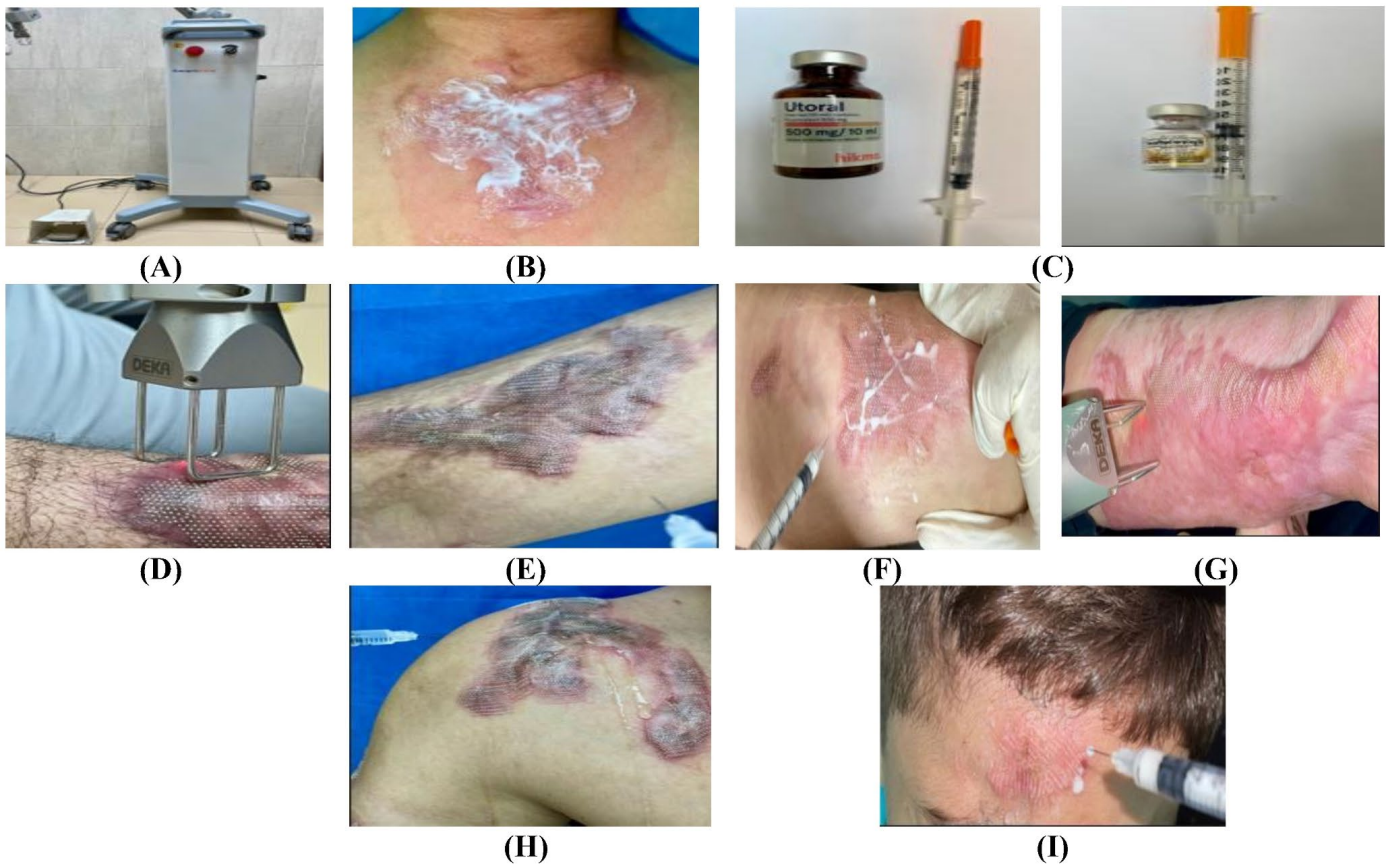
(**A**) Fractional ablative CO_2_ 10,600-nm laser (DEKA, smartXide, Italy), (**B**) Topical applying of anesthetic cream (Paridocaine cream) 60 min before the laser session, (**C**) Preparing the 5-fluorouracil solution and the triamcinolone acetonide solutions, (**D**) Applying a fractional ablative carbon dioxide laser session, Topical applying of (**E**) 5-fluorouracil solution after the laser session, (**F**) Triamcinolone acetonide solution rubbing after the laser session, (**G**) Group A: a patient treated with fractional CO_2_ laser only, (**H**) Group B: a patient treated with fractional CO_2_ laser and topically applied 5-FU, (**I**) Group C: a patient treated with fractional CO_2_ laser and topically applied triamcinolone acetonide

Post-treatment care included applying silicone gel after the session and twice daily for the entire study for 6 months, applying moisturizer 3 times a day until healing, and using daily sunscreen (SPF 50+). Patients were advised to avoid direct sunlight and warned not to rub, scratch, or peel the crust or epidermal debris abruptly. Figure 1.

Patients were followed up every month for six months. At every visit, the Vancouver Scar Scale (VSS) and the Patient Scar Assessment Scale were used to assess scar features and patient satisfaction [11, 12]. The primary endpoint was the objective assessment by the Vancouver Scar Scale (VSS), and the secondary endpoints included the subjective evaluation of the patients’ improvements in patient-reported symptoms (itching and pain) and satisfaction, assessed four weeks after the final CO₂ laser session. By January 2024, each participant had finished six therapy sessions. The clinical data collected included demographics, burn injury specifics, VSS scores, scar appearance, and laser treatment parameters.

## Statistical analysis

The statistical analysis was carried out using the SPSS v26 program (IBM Inc., Chicago, Illinois, United States of America). A comparison of the quantitative variables across the three groups was conducted by employing an analysis of variance (ANOVA) test in conjunction with a post hoc test (Tukey). The statistical measures used to express the measurements were the mean and the standard deviation (SD). The Chi-square test was also applied to conduct an analysis of the qualitative variables, which were supplied in the form of frequency and percentage (%). To be regarded as statistically significant, it was necessary to have a two-tailed P value that was lower than 0.05.

## Results

**Fig. 2:**
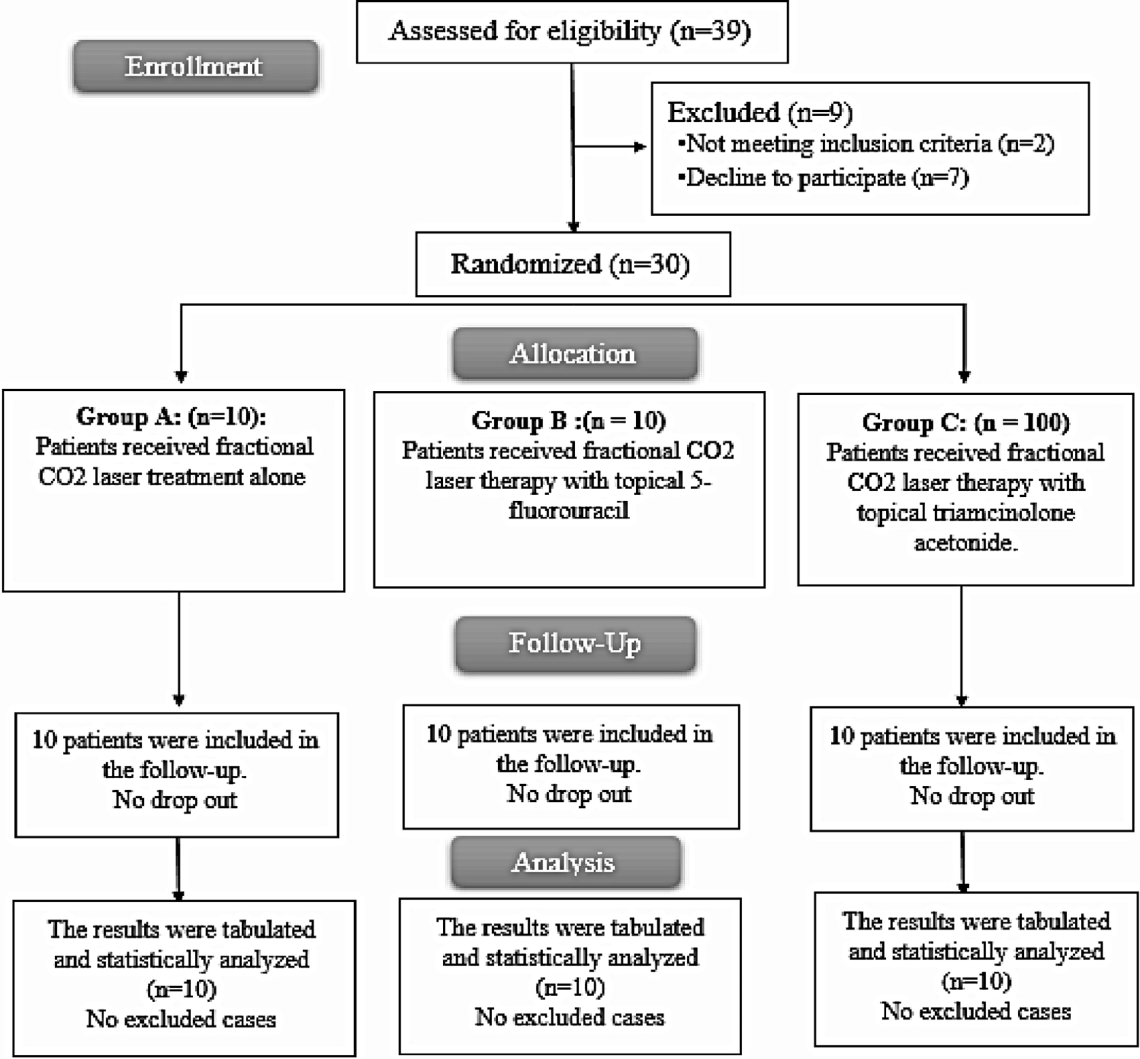
CONSORT flow chart of the studied patients

Thirty-nine patients were assessed for eligibility; two did not meet the criteria, and seven refused to participate in the study. The remaining 30 patients were randomly allocated into three equal groups (10 patients each). All allocated patients were followed up and analysed statistically. Figure 2.

No statistically significant differences were observed among the three study groups regarding demographic and clinical characteristics (Table 1).

**Table 1:** Comparison between the three studied groups according to demographic data and clinical data

		Group A (*n* = 10)	Group B (*n* = 10)	Group C (*n* = 10)	*P*
Age (years)		25.90 ± 11.98	28.30 ± 12.34	27.40 ± 10.66	0.898
Sex	Male	4(40.0%)	5(50.0%)	6(60.0%)	0.670
	Female	6(60.0%)	5(50.0%)	4(40.0%)	
Clinical data					
Site of scar	Extremities	4(40.0%)	4(40.0%)	8(80.0%)	0.254
	Acral	1(10.0%)	2(20.0%)	0(0.0%)	
	Trunk	5(50.0%)	4(40.0%)	2(20.0%)	
Duration		5.20 ± 1.40	4.60 ± 1.43	4.50 ± 1.43	0.501
Family history		4(40.0%)	3(30.0%)	3(30.0%)	1.000

Data are presented as mean ± SD or frequency (%)

Following the results for each group individually: In Group A, no significant improvement in VSS parameters was noted following treatment. In Group B, significant improvements were observed in height, pliability, and scar vascularity, with a pre- and post-treatment difference (*P* < 0.05). In Group C, significant enhancements were identified in height, pliability, and pigmentation after treatment (*P* < 0.05) (Table 2).

**Table 2:** Comparison between pre and post according to VSS in Group A, B, and C

	Pre	Post	*p*
**Group A**			
Vascularity	1.30 ± 1.06	1.10 ± 0.74	0.317
Pliability	2.40 ± 0.70	1.80 ± 1.14	0.058
Height	2.30 ± 0.48	2.0 ± 0.67	0.180
Pigmentation	1.0 ± 1.05	1.40 ± 0.97	0.339
VSS	7.10 ± 2.28	6.30 ± 2.41	0.263
**Group B**			
Vascularity	1.40 ± 1.17	0.40 ± 0.52	**0.047** ^*^
Pliability	2.60 ± 0.52	0.90 ± 0.32	**0.004** ^*^
Height	2.60 ± 0.52	1.40 ± 0.52	**0.006** ^*^
Pigmentation	1.40 ± 0.97	0.60 ± 0.84	0.071
VSS	8.0 ± 2.54	3.30 ± 0.95	**0.005** ^*^
**Group C**			
Vascularity	1.50 ± 1.18	0.50 ± 0.53	0.065
Pliability	2.60 ± 0.52	1.20 ± 0.79	**0.004** ^*^
Height	2.50 ± 0.71	1.0 ± 0.94	**0.004** ^*^
Pigmentation	1.30 ± 0.82	0.50 ± 0.71	**0.046** ^*^
VSS	7.90 ± 2.13	3.20 ± 1.93	**0.005** ^*^

Data is presented as mean ± SD. * Significant p-value < 0,05, VSS: Vancouver Scar Scale

Before the initiation of treatment, all values of the Vancouver Scar Scale (VSS) were comparable across the three groups, with no statistically significant differences observed. After the treatment, height and total VSS scores demonstrated statistically significant differences among the three groups (*P* < 0.05). The analysis revealed significant improvements when comparing Group B with Group A and Group C with Group A. Conversely, no statistically significant differences were observed between Group B and Group C (Table 3).

**Table 3:** Comparison between the three studied groups according to VSS

		Group A (*n* = 10)	Group B (*n* = 10)	Group C (*n* = 10)	*P*
Pre	Vascularity	1.30 ± 1.06	1.40 ± 1.17	1.50 ± 1.18	0.938
	Pliability	2.40 ± 0.70	2.60 ± 0.52	2.60 ± 0.52	0.778
	Height	2.30 ± 0.48	2.60 ± 0.52	2.50 ± 0.71	0.406
	Pigmentation	1.0 ± 1.05	1.40 ± 0.97	1.30 ± 0.82	0.573
	VSS	7.0 ± 2.26	8.0 ± 2.54	7.90 ± 2.13	0.585
**Post**	Vascularity	1.10 ± 0.74	0.40 ± 0.52	0.50 ± 0.53	0.057
	Pliability	1.80 ± 1.14	0.90 ± 0.32	1.20 ± 0.79	0.119
	Height	2.0 ± 0.67	1.40 ± 0.52	1.0 ± 0.94	**0.035** ^*^
	Pigmentation	1.40 ± 0.97	0.60 ± 0.84	0.50 ± 0.71	0.071
	VSS	6.30 ± 2.41	3.30 ± 0.95	3.20 ± 1.93	**0.003** ^*^
	Sig. bet. Grps	**P**_**1**_ = 0.005^*^, **P**_**2**_ = 0.002^*^, **P**_**3**_ **= 0.784**			

Data is presented as mean ± SD. * Significant p-value < 0,05, p1: p-value for comparing Group A and Group B, p2: p-value for comparing Group A and Group C, p3: p-value for comparing Group B and Group C, VSS: Vancouver Scar Scale

The evaluation of pain, pruritus, and overall patient satisfaction across the three groups following treatment revealed no statistically significant differences in pain or pruritus. However, overall patient satisfaction showed a statistically significant difference among the groups (*P* < 0.05). Post hoc analysis revealed a notable improvement in satisfaction when comparing Group B with Group A and Group C with Group A. In contrast, the difference between Group B and Group C was not statistically significant. Regarding the complications: In Group A, no complications were reported apart from transient pain experienced by all patients during laser treatment and in all three groups. In Group B, systemic complications commonly associated with 5-fluorouracil (5-FU), such as anemia, leukopenia, and thrombocytopenia, were absent. Nevertheless, local adverse effects were frequently observed, including burning sensation, ulcerations, and hyperpigmentation. In Group C, triamcinolone acetonide (TAC) was associated with side effects such as telangiectasia, erythema, and skin atrophy (Table 4).

**Table 4:** Comparison between the three studied groups according to different parameters and complications

	Group A (*n* = 10)	Group B (*n* = 10)	Group C (*n* = 10)	*P*
Pain	3.50 ± 1.27	3.0 ± 1.25	2.80 ± 0.42	0.327
Pruritus	3.0 ± 1.33	2.60 ± 0.84	2.90 ± 1.20	0.721
Overall patient satisfaction	5.80 ± 1.32	7.70 ± 0.67	7.0 ± 0.94	**0.001** ^*^
Sig. bet. grps.	**P**_**1**_ = 0.001^*^, **P**_**2**_ = 0.034^*^, P_3_ = 0.286			
**Complications**				
Complications	0(0.0%)	8(80.0%)	4(40.0%)	0.002*
Ulceration	0(0.0%)	4(40.0%)	0(0.0%)	0.023*
Hyperpigmentation	0(0.0%)	3(30.0%)	0(0.0%)	0.086
Burning	0(0.0%)	6(60.0%)	1(10.0%)	0.008*
Atrophy	0(0.0%)	0(0.0%)	1(10.0%)	1.000
Telangiectasia	0(0.0%)	0(0.0%)	2(20.0%)	0.315

Data are presented as mean ± SD. * Significant p-value < 0,05, p1: p-value for comparing between Group A and Group B, p2: p-value for comparing between Group A and Group C, p3: p-value for comparing between Group B and Group C

**Fig. 3:**
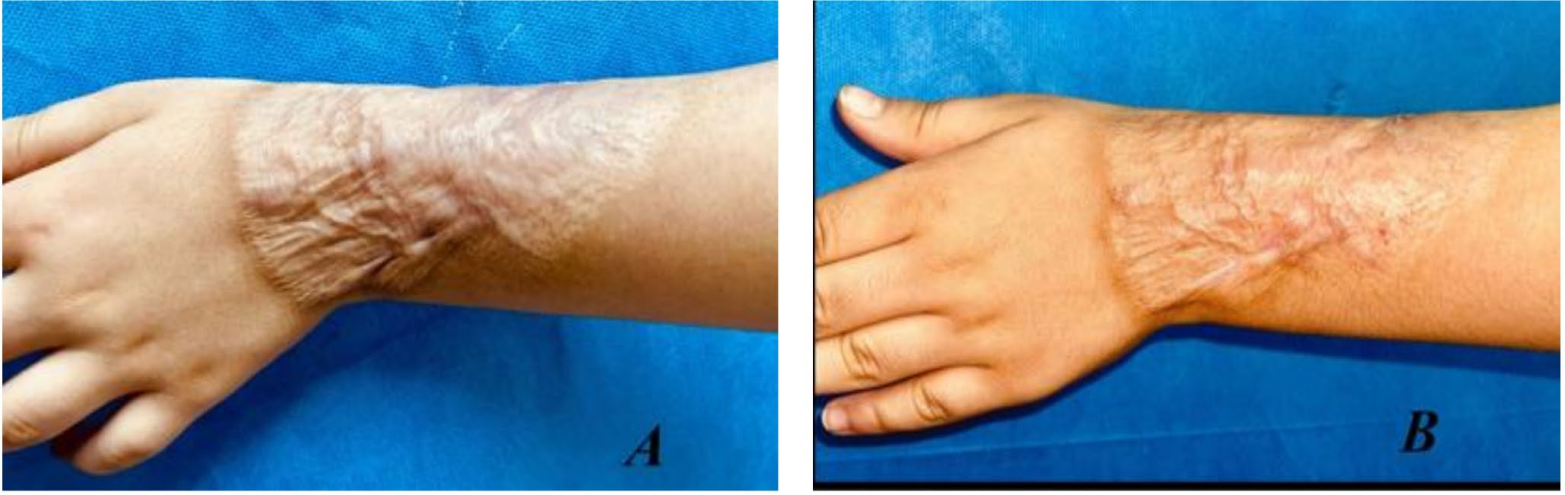
(**A**) Before and (**B**) after treatment with fractional CO_2_ laser for 6 months

**Fig. 4:**
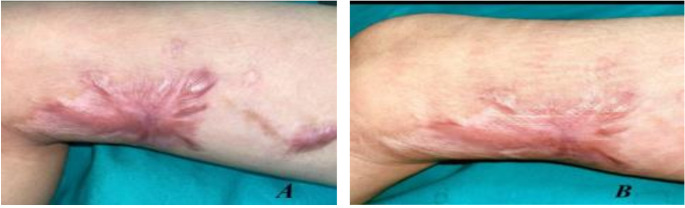
(**A**) Before and (**B**) after treatment with fractional CO_2_ laser for 6 months

**Fig. 5:**
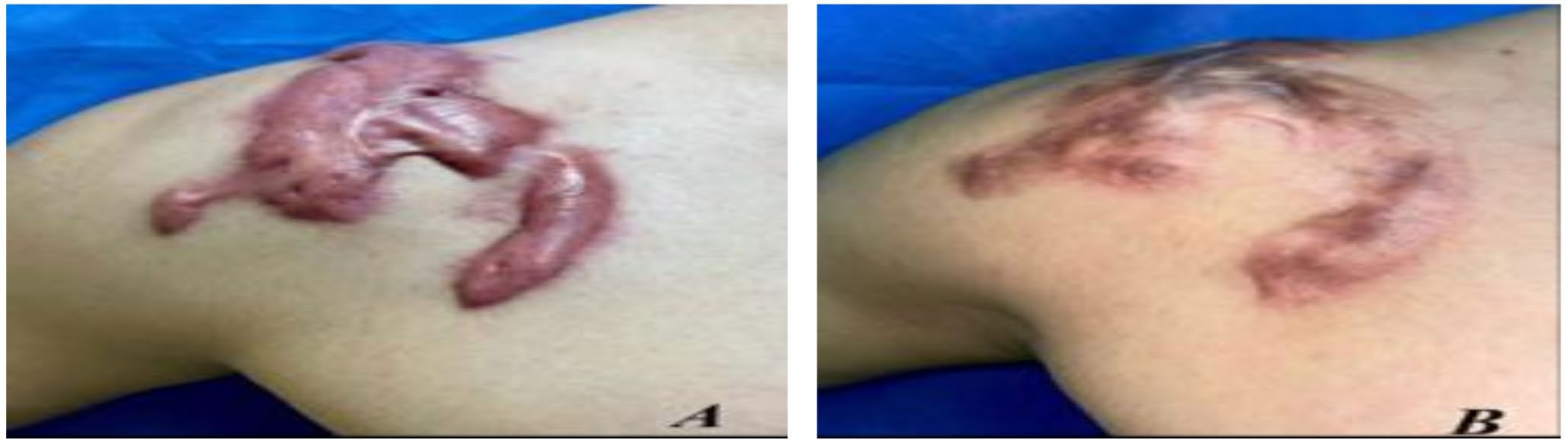
(**A**) Before and (**B**) after treatment with fractional CO_2_ laser for 6 months and topically applied 5-FU for 6 months

**Fig. 6:**
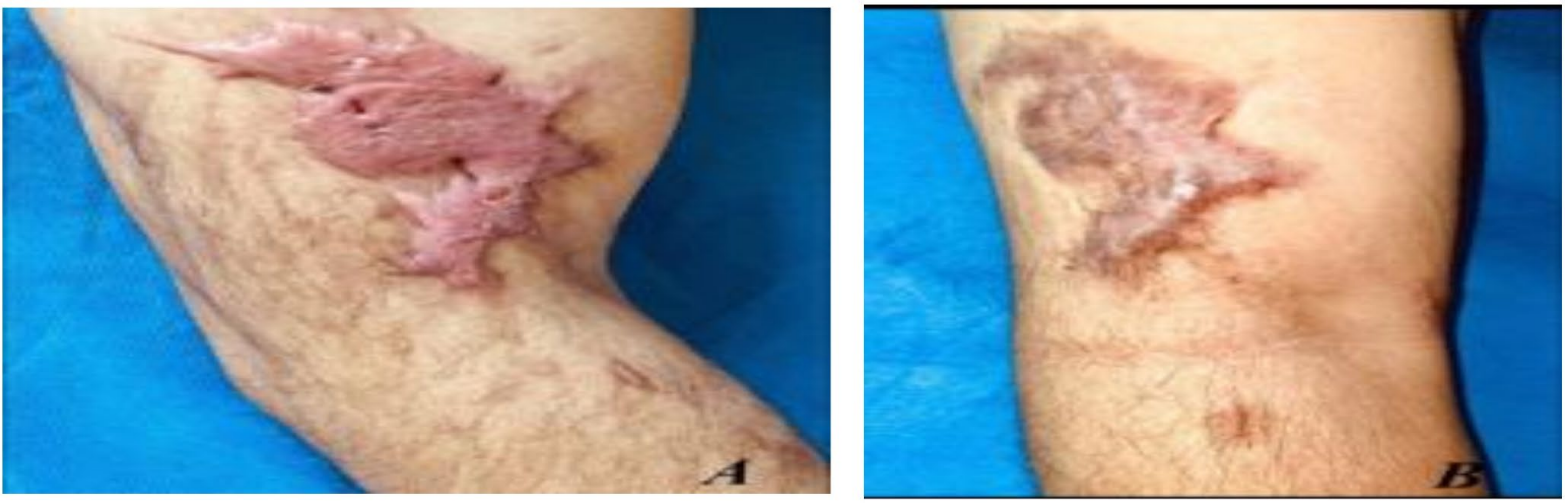
(**A**) Before and (**B**) after treatment with fractional CO_2_ laser for 6 months and topically applied 5-FU for 6 months

**Fig. 7:**
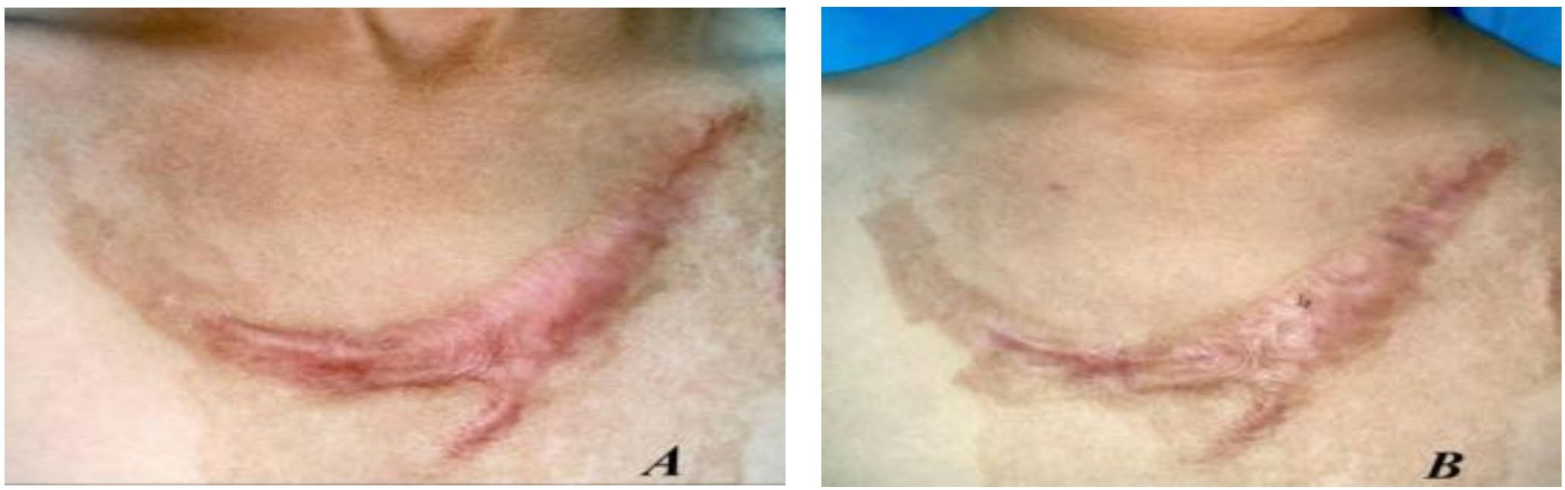
(**A**) Before and (**B**) after treatment with fractional CO_2_ laser for 6 months and topically applied Triamcinolone acetonide for 6 months

**Fig. 8:**
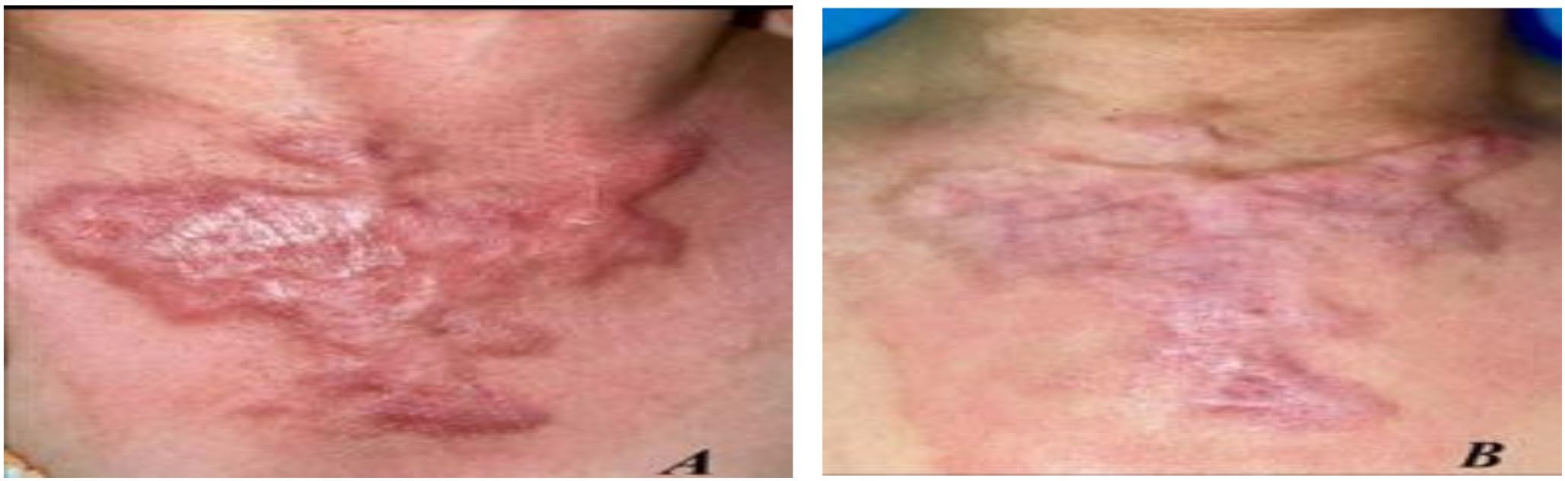
(**A**) Before and (**B**) after treatment with fractional CO_2_ laser for 6 months and topically applied Triamcinolone acetonide for 6 months

**Group A**,

### Case 1

Female patient with a 4-month postburn hypertrophic scar on her left forearm. Figure 3.

### Case 2

A female patient with a 5-month postburn hypertrophic scar on her right knee. Figure 4.

**Group B**,

### Case 1

A male patient with a 3-month postburn hypertrophic scar on his right shoulder. Figure 5.

### Case 2

a 6-month postburn hypertrophic scar on his right arm. Figure 6.

**Group C**,

### Case 1

A female patient with a 6-month postburn hypertrophic scar on her chest. Figure 7.

### Case 2

A female patient with a 3-month postburn hypertrophic scar on her chest. Figure 8.

## Discussion

HTSs are characterized as elevated, visible scars that usually regenerate independently and do not expand into the surrounding tissues. These scars have the following characteristics: continuous inflammation and fibrosis, dermal tissue proliferation, and persistent excessive deposition of extracellular matrix (ECM) proteins, primarily collagen, from fibroblasts [13, 14].

The study findings demonstrate that both TAC and 5-FU are effective in managing HTSs. Combining fractional ablative CO_2_ and topical application of 5-FU solution or triamcinolone acetonide solution is more effective than fractional ablative CO_2_ alone in HTSs management.

Aligned with our findings, Waibel et al. [15] utilized laser-assisted drug delivery of triamcinolone acetonide and 5-fluorouracil to treat hypertrophic scars, and we adopted their recommended dosing regimens as described earlier. While their study involved 20 patients undergoing three treatment sessions and evaluated outcomes using scar height and surface area, our study expanded on this work by including 30 patients, divided into three groups, who received six treatment sessions. Additionally, we employed the Vancouver Scar Scale (VSS) and the Patient and Observer Scar Assessment Scale (POSAS) for a more comprehensive evaluation of scar improvement.

To assess the independent effect of the laser, Group A was used as a control group without adjunctive drug therapy. Consistent with previous findings, our study observed no significant differences in scar area reduction between TAC and 5-FU. Similarly, Waibel et al. [16] reported using laser-assisted corticosteroid delivery in 15 patients with hypertrophic scars of varying etiologies to assess improvement with a modified Manchester Quartile Score. Unlike their investigation, which focused on a broader range of scar causes and used a single scoring system, our study specifically targeted post-burn hypertrophic scars and employed VSS for detailed evaluation and comparisons among groups to isolate the effects of individual treatments. Their findings demonstrated overall improvement, supporting the efficacy of laser-assisted drug delivery in hypertrophic scar management.

Our study specifically targeted early hypertrophic scars within six months of their development, as these scars are known to respond more effectively to laser modulation due to the active involvement of inflamatory and growth factors during early scar maturation. In contrast, many prior studies primarily focused on older scars. For instance, El-Zawahry et al. [17] reported a mean scar duration of 8.1 ± 9.4 years, while Azzam et al. [18] examined scars with a duration range of up to 19.83 years.

Additionally, our study exclusively focused on post-burn hypertrophic scars, providing a more focused analysis of scars with a specific etiology and duration. In comparison, Azzam et al. included both hypertrophic scars and keloids from various causes. El-Zawahry et al. [17] studied thermal scars with a broader inclusion of older scars and keloids. This narrower scope in our research enhances its specificity and applicability for treating early post-burn hypertrophic scars, distinguishing it from the broader focus of previous studies. Our research was performed using clinical evaluations of VSS and PSOAS scores.

Haak et al. [19] observed that the ablation depth had no significant impact on drug delivery. This indicates that once the stratum corneum’s epidermal barrier is disrupted, drugs diffuse through the dermis similarly, regardless of the depth of the microthermal ablation zones.

Our study couldn’t assess this, as the treatment parameters were categorized into low and high parameters at different scar heights. So, it is important to ask whether laser depth optimized the delivery by changing the parameters, but it will need to be investigated for each drug at the same height with different parameters.

Consistent with our research, a study by Cavalié et al. [20] was conducted on 23 patients with keloid scars who used laser-assisted drug delivery with topical corticosteroid. However, their protocol differed from ours as they use topical betamethasone cream, and our formula is the solution. In a study by Issa et al. [21], four patients with HTSs underwent fractional ablative laser therapy and then applied a 20 mg/ml topical TAC spray while gently rubbing their affected areas; this approach aligns closely with our Group C protocol. Their results proved the remarkable efficacy of steroids LADD and rapid scar resolution. This gave us the idea that the solution formula is more applicable to controlling all patients’ doses and drug distribution.

Zhuang Z et al. [22] performed a meta-analysis comparing triamcinolone acetonide (TAC), 5-fluorouracil (5-FU), 5-FU + TAC, and verapamil for healing keloids and hypertrophic scars, assessing outcomes such as scar height, vascularity, pliability, pigmentation, and total scores on the VSS or POSAS. Unlike our work, which utilized laser-assisted drug delivery (LADD), their method relied on intralesional injections. They found that while TAC showed short-term effects, 5-FU, or its combination with TAC or verapamil, was more effective in the medium- to long-term. TAC was associated with a greater risk of skin atrophy and telangiectasia. The study’s drawbacks, including a small sample size, show the need for more extensive, diverse studies. Their results support the growing evidence favoring 5-FU in pathological scar management, aligning with the new LADD methods studied in our research.

In disagreement with our study, Prince et al. [23] stated that the use of 5- FU in different dermatological disorders and skin tumors but not the HTSs like ours. In corresponding with us, they mentioned the most common side effects of 5- FU, such as mild erythema, hyperpigmentation, and ulcerations. As confirmed by the literature, ulceration is a common side effect of intradermal 5-FU use in hypertrophic scars and keloids [24]. On the other hand, the use of 5-FU as laser-assisted drug delivery is safe with no ulceration, as described by other studies [24]. Unfortunately, four patients complained about superficial ulceration after one session during their course of treatment. This was due to scratching, as they had burning and itching sensations after the session. These ulcerations lasted less than two weeks and were managed by fusidic acid ointment 2%. In the following sessions, antihistaminic antiallergic medication (chlorphenoxamine HCL) was applied after the session, which alleviated the burning and itching and prevented further scratching and ulceration.

The limitations of this study include the fact that the sample size of 30 patients was recruited from a single clinical site. Recruitment of more study participants in future studies will strengthen the power of the findings, which may include a demonstration of statistically significant variations in scars after the different modalities. Furthermore, future studies may seek to evaluate the combination of topical regimens comprising both 5-FU and triamcinolone with laser-assisted administration of either medication alone versus other drugs. An approach that has been followed in prior intralesional trials with the inclusion of topical-only groups as controls. Nevertheless, the findings enhance a growing body of data supporting using 5-fluorouracil to manage pathologic scars.

## Conclusions

Although there was no statistically significant difference between the effects of 5-fluorouracil and TAC, indicating that neither medication is more effective, the HTSs of this trial showed improvements in pliability and pigmentation and reductions in overall scar surface area and thickness. Nevertheless, the administration of 5-fluorouracil did not promote the occurrence of cutaneous atrophy or the formation of telangiectasia, which are frequently observed as side effects of corticosteroid therapy. The unfavorable effects observed may be attributed to the laser’s ability to increase the amount of corticosteroid the body can absorb. While this method enables direct medication administration to the scar tissue, it also reduces the distance the corticosteroid needs to travel to reach the deeper layers of the skin. However, 5-fluorouracil has been found to induce side effects such as ulceration, burning, and hyperpigmentation. Therefore, it is advisable to use it with caution.

## Data Availability

Data is available upon reasonable request from corresponding author.
